# Dataset of Smartphone-Based Finger Tapping Test

**DOI:** 10.1038/s41597-024-04052-y

**Published:** 2024-11-21

**Authors:** Ramon Costa Lima, Felipe André da Costa Brito, Rodrigo Luz do Nascimento, Sthephanie Nazaré e Silva Martins, Luis Carlos Monteiro Pereira, Jéssica Portal Seabra, Hugo Leonnardo Chaves Farias, Laenna Morgana Cunha da Silva, Victor Matheus Silva de Miranda, Anderson Belgamo, André dos Santos Cabral, Bianca Callegari, Anselmo de Athayde Costa e Silva, Alex Crisp, Cândida Helena Lopes Alves, Eliza Maria da Costa Brito Lacerda, Givago Silva Souza

**Affiliations:** 1 Instituto de Ciências Biológicas, Universidade Federal do Pará, Belém, Brazil; 2 Núcleo de Teoria e Pesquisa do Comportamento, Universidade Federal do Pará, Belém, Brazil; 3 Instituto de Ciências da Saúde, Universidade Federal do Pará, Belém, Brazil; 4 Universidade Federal do Oeste do Pará, Santarém, Brazil; 5 Instituto Federal de São Paulo, Piracicaba, Brazil; 6 Centro de Ciências Biológicas e da Saúde, Universidade do Estado do Pará, Belém, Brazil; 7 Instituto de Ciências da Educação, Universidade Federal do Pará, Belém, Brazil; 8 Univerisidade Federal do Maranhão, São Luís, Brazil; 9 Núcleo de Medicina Tropical, Universidade Federal do Pará, Belém, Brazil

**Keywords:** Predictive markers, Neural ageing

## Abstract

The finger tapping test (FTT) is a neuropsychological test that measures motor speed and coordination. It involves tapping a designated surface with a specific finger as quickly as possible for a certain duration. Touchscreen of smartphones has been used as interface to record the tap, what enables to extract information about the taps. The present study represents an initiative of construction for a national Database of Smartphone-Based FTT, which includes data from 176 healthy male and female adults ranging in age from 18 to 74 years. Participants were asked to perform the FTT using one or both hands, tapping on a central area of a smartphone as many times as possible within a 30-second interval. Data were extracted using an Android application, encompassing details such as touch timing, spatial coordinates, sex, smartphone model, hand used, and age. A Python-developed web visualization tool for individual and averaged analysis. This database serves as informative foundation for a healthy adult sample and supports further exploration and international comparative analyses of FTT performance.

## Background & Summary

The Finger Tapping Test (FTT) is a classical motor function assessment in which an individual is required to tap at the highest possible frequency on a surface^1^. Over the years, it has undergone several adaptations based on the available technology of each era^2–4^. In its early stages, the test involved tapping on a telegraph key, and subsequently, it transitioned to using computer keyboard keys, mice, accelerometers, and image capture technologies. In recent years, with the widespread accessibility of smartphones and their touch-sensitive screens, a multitude of studies have sought to utilize smartphones for the administration of the FTT^5–7^.

The use of smartphones for FTT enables to keep the temporal analysis of the classical approach of the test, but it adds new dimensions to the analysis such as the spatial domain, that already have been described as significant variable to distinguish healthy and people with Parkinson’s disease^5^.

While various investigations utilizing smartphones for FTT assessment have been conducted in different countries, there is currently no established population norm or comparative analysis between populations, even considering cultural and anthropometric variations across countries. This study represents an initiative of establishment of a national database for the smartphone-based FTT. Within this database, there are information about sex, handedness, age, and smartphone devices used for the test. Some of these data were previously published^7^.

### Related work

Prior to the digital revolution in healthcare, the Finger Tapping Test (FTT) was widely employed for motor impairment identification in various diseases and for monitoring the effectiveness of therapies^8–10^. The utilization of touch-sensitive screens on smartphones for FTT execution provides a significant opportunity for test applicability due to the widespread availability of smartphones. However, the abundance of programming tools for smartphones introduces methodological variations in studies employing smartphones for the FTT, necessitating careful consideration when comparing results across different studies. Diverse approaches have been observed in these studies, with some resembling classical FTT tests where motor performance is assessed through taps in a single area, typically the center of the smartphone screen^7^. Conversely, other studies involve evaluating alternating taps in neighboring locations on the screen^7,9^. Some studies use touch buttons on the screen^9^, while others recognize touches anywhere on the screen^7^. These methodological differences may impact data extraction, particularly concerning the spatial distribution of touches on the screen, a factor that can only be accurately extracted if touch locations are registered by the smartphone’s touch-sensitive screen.

### Our dataset and potential uses

The present database encompasses a collection of data related to the smartphone-based Finger Tapping Test (FTT) from a sizable Brazilian sample residing in urban areas in the northern region of the country. All participants in the database are currently right-handed, spanning various age groups, and do not exhibit clinical complaints or diagnoses of chronic or degenerative diseases. This database holds potential utility as a reference group for clinical comparisons in both scientific studies and routine clinical assessments. The data was obtained from Protocol I of the FFT running using Momentum Touch application, which closely mirrors the classical FTT approach and records the timing and coordinates of touchscreen taps.

## Methods

### Participants

The dataset comprises 176 healthy adult participants, aged between 18 and 74 years. All participants were questioned about hand complaints and the use of any medication. No participant included in the present dataset reported osteoarthritis, any other chronic disease, or even the use of medication during the testing period that could affect the motricity of the limbs. All participants were considered healthy for inclusion in the dataset. All participants reported no clinical complains about motricity function, no history of neurodegenerative diseases, as well as all participants had no clinical complaints regarding hand function. All participants willingly agreed to be part of the research sample after a detailed explanation of the procedures, which received approval from the Research Ethics Committee of the Federal University of Pará (report #6.036.494). The informed consent process was conducted in writing, ensuring a comprehensive understanding of the research objectives and procedures before participants chose to participate.

### Experimental protocol, and instrumentation

**Fig. 1:**
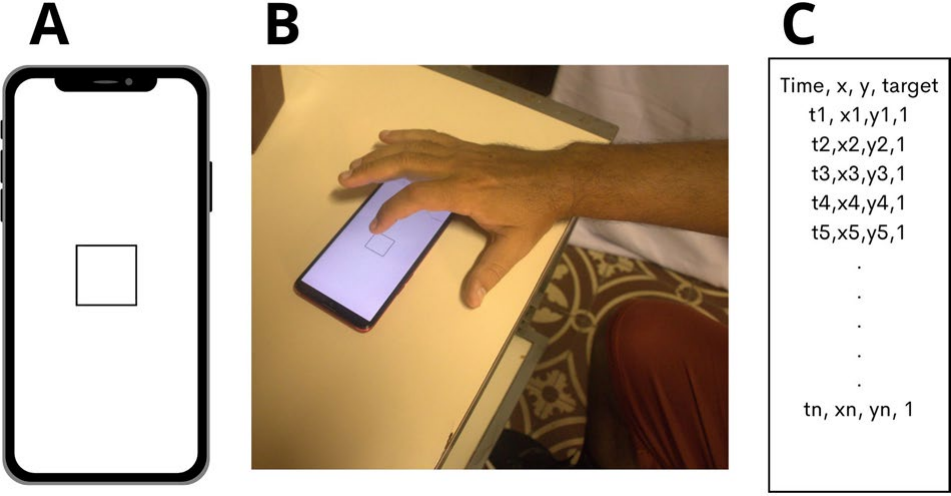
Procedure of testing. (**A**) Screen design during testing: The participant was required to tap as quickly as possible in the central area of the screen. (**B**) Hand positioning during the test: The smartphone was placed in front of the participant, who kept their hand and wrist pressed against the table while the index finger tapped the screen. (**C**) Arrangement of the output file from the FTT app.

The Momentum Touch Android application was employed for test execution, utilizing various smartphone models for the assessment. The Finger Tapping Test (FTT) is composed by three test protocols. For the present study, we used only the Protocol I, wherein participants were instructed to tap a central area using the index finger as many times as possible within a 30-second interval^7^. The procedure of this protocol is closer to the classical FTT which it carried out tapping a physical key or surface. The participants were seated in front of a table, and the smartphone was placed on the table in landscape orientation in front of them. The participant’s wrist had to remain in contact with the table, and the index finger of the hand being tested touched the screen. After a visual cue on the smartphone screen, the participant tapped solely with the index finger on the central area of the screen. Momentum app exports a text file with 4 columns (moment of the tap, x coordinate of tap, y coordinate of tap, logical indicator (Fig. 1).

## Data Records

**Fig. 2:**
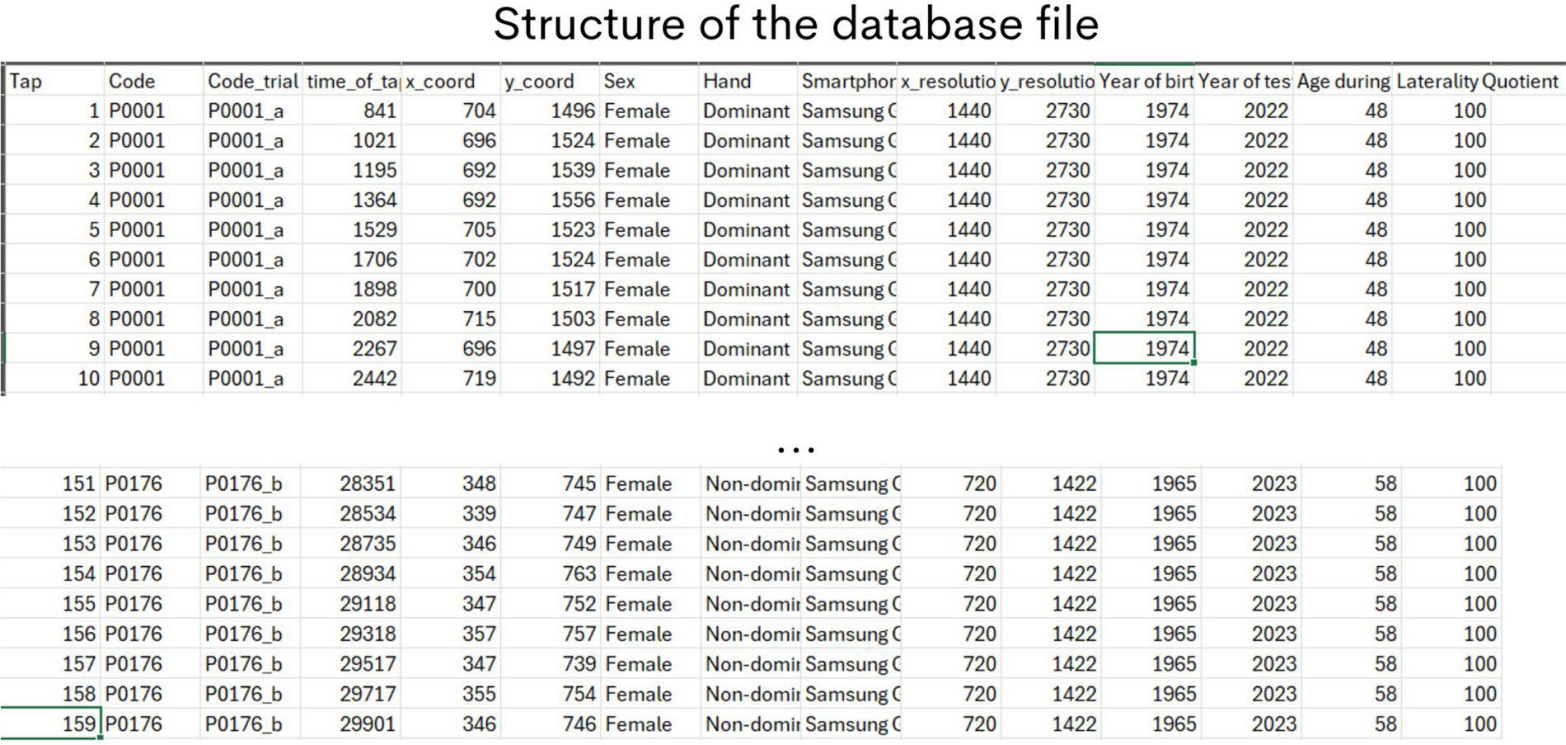
Structure of the database file. Tap column shows the tap number of an individual test. Code column indicates the row for an individual participant. The individual FTT test information is separated into time of taps, spatial x coordinates, spatial y coordinates columns. The Code_trial indicate the rows containing the information of the same individual test. Code is the information for an individual participant. Sex, hand, Smartphone, x_resolution, y_resolution Year of the birth, Year of the test, and Age during the test columns brings the information indicated by the column name.

The database consists of one CSV file containing columns with information about the participant and the smartphone used for the test (number of the tap, code, code_trial, moment of the tap, x coordinate of the tap, y coordinate of the tap, sex, handedness, smartphone model, x screen resolution, y screen resolution, year of birth, year of the test, participant’s age at the test, and laterality quotient laterality quotient from Edinburg Handedness Inventory – short form). The data from each single test is represented by the single code_trial and each row bring time and spatial information from the performance during the test. The file is available in the supplementary materials. The structure of the database file is shown in the Fig. 2. The datasets for smartphone-based FTT described in this paper is available in Figshare^11^. An application for web visualization is also available at https://brazilianfinger-tapping-database.streamlit.app. In the web application the reader will contact with kinematic parameters extracted from the smartphone-based finger tapping test such as number of taps, inter tapping interval, maximum inter tap interval, minimum inter tap interval, variability of the inter tap interval, total spatial deviation, and the ellipse covering 95% of the spatial distribution.

## Technical Validation

The data provided in the present study was collected using commercial smartphones and touchscreens with proprietary technical validation. Hence, we do not provide any test technical validation datasets. All the data was visually inspected by trained researchers. We acknowledge the importance of technical validation to ensure data quality and minimize error. In the present dataset, our focus was on collecting exploratory data to understand general patterns rather than establishing a highly controlled dataset, which is why technical validation experiments were not conducted initially. However, we recognize that technical validation is crucial for the broader application of our results. As a next step, we plan to conduct additional technical validation experiments using a representative subset of the dataset to proceed concurrent validity using traditional tapping device or systems based in motion capture or accelerometers. Furthermore, we will explore advanced statistical techniques to assess the consistency and robustness of our data more rigorously. These steps will be essential to enhance the reliability of the dataset and improve the overall quality of our analyses, thereby minimizing potential sources of error.

### Limitations

It was not possible to collect information regarding the participants’ level of education and expertise in using digital devices. The size of this data is not sufficient to answer a research question such as establishing the reference values of finger tapping performance in healthy subjects stratified by age groups.

## Data Availability

Python routines to process the dataset is freely available on the following repository: https://github.com/givagosouza2/fingertappingdatabase.
